# Size matters—in vitro behaviour of human fibroblasts on textured silicone surfaces with different pore sizes

**DOI:** 10.1007/s10856-020-6360-5

**Published:** 2020-02-03

**Authors:** Julia Tolksdorf, Raymund E. Horch, Jasmin S. Grüner, Rafael Schmid, Annika Kengelbach-Weigand, Dirk W. Schubert, Siegfried Werner, Dominik Schneidereit, Oliver Friedrich, Ingo Ludolph

**Affiliations:** 1 Department of Plastic and Hand Surgery and Laboratory for Tissue Engineering and Regenerative Medicine, University Hospital of Erlangen Friedrich-Alexander-University of Erlangen-Nürnberg (FAU), Krankenhausstraße 12, 91054 Erlangen, Germany; 2 Institute of Polymer Materials, Friedrich-Alexander-University of Erlangen-Nürnberg (FAU), Martensstrasse 7, 91058 Erlangen, Germany; 3 Institute of Medical Biotechnology, Friedrich-Alexander-University of Erlangen-Nürnberg (FAU), Paul-Gordan-Str. 3, 91052 Erlangen, Germany

## Abstract

Capsular contracture remains a challenge in plastic surgery and represents one of the most common postoperative complications following alloplastic breast reconstruction. The impact of the surface structure of silicone implants on the foreign body reaction and the behaviour of connective tissue-producing cells has already been discussed. The aim of this study was to investigate different pore sizes of silicone surfaces and their influence on human fibroblasts in an in vitro model. Four different textures (no, fine, medium and coarse texture) produced with the salt-loss technique, have been assessed in an in vitro model. Human fibroblasts were seeded onto silicone sheets and evaluated after 1, 4 and 7 days microscopically, with viability assay and gene expression analysis. Comparing the growth behaviour and adhesion of the fibroblasts on the four different textures, a dense cell layer, good adhesion and bridge-building ability of the cells could be observed for the fine and medium texture. Cell number and viability of the cells were increasing during the time course of experiments on every texture. *TGFß1* was lowest expressed on the fine and medium texture indicating a trend for decreased fibrotic activity. For silicone surfaces produced with the salt-loss technique, we were able to show an antifibrotic effect of smaller sized pores. These findings underline the hypothesis of a key role of the implant surface and the pore size and pore structure in preventing capsular contracture.

## Introduction

Capsular contracture (CC) remains a challenge in plastic surgery and represents one of the most common postoperative complications following alloplastic breast reconstruction or breast augmentation associated with revisional surgery and implant replacement [1, 2]. Various theories exist on the aetiology of CC but the exact causes are still unknown [3–5]. Several in vitro and in vivo studies have investigated a possible prophylactic influence of different drugs or modifications of the implants providing no distinct evidence of effectiveness in the past [6]. This includes the administration of Simvastatin, Collagenase or Triamcinolone into capsular tissue and the coating of implants with antibiotics, spider silk, Montelukast or Triamcinolone [7–20].

Beside factors like the anatomic implant location, a subclinical infection or inflammation, bacterial biofilm, radiotherapy of the breast, material properties of the implant surface have been discussed to play a role in the development of CC [4, 5, 21–25]. Depending on different texturing techniques (e.g. salt-loss, imprinted, secondarily coated) and the surfaces created (textured, microtextured, smooth), the incidence of CC is reported with approximately 16% [26].

A common property of synthetic materials, like silicone implants, is the potential induction of adverse immune reactions resulting in fibrotic encapsulation, inflammation, impairment of healing, tissue destruction, or even isolation and rejection of medical devices [27]. Excessive capsule formation identified as CC in breast implants manifests itself with a painful tightening and hardening of the capsule surrounding the implant. To define the stage of the CC in breast implant patients, the modified form of the Baker clinical grading system is commonly used today [28].

As the surface structure of silicone implants has been repeatedly discussed as a major factor to influence the severity of the foreign body reaction, current investigations focus on different surface textures and their texturizing techniques as well as their influence on the behaviour of connective tissue cells and the extent they contribute to CC [3, 17, 22, 29–31]. Recently, this aspect has also gained attention due to a potential connection between surface texture and the occurrence of the so-called breast implant-associated anaplastic large cell lymphoma (BIA-ALCL), a rare type of Non-Hodgkin’s lymphoma. Textured implants have been accused to pose a higher risk for patients to develop BIA-ALCL, but it has also been described to occur with smooth surface implants [32]. However, in most of the cases it remains unclear if previous smooth implants had been used in the same patients before textured implants were inserted since the database of reported BIA-ALCL cases is quite inhomogeneous.

Rough-textured implants are preferred to smooth ones due to their lower association with CC, especially in combination with a sub-muscular implantation [22, 23, 31, 33–35]. In this context, different surface textures could alter the host’s response to the integration of the foreign material, so that tissue ingrowth may produce a host prosthesis interface that is more stable and compatible [29, 36, 37].

To gain more insights into possible biological behaviours of silicone implant surfaces, the current preliminary in vitro study concentrates on the question to what extent different pore sizes, fabricated with the salt-loss technique, influence the behaviour of fibroblasts at silicone surfaces.

## Material and methods

### Silicone

The textured surfaces were produced with the salt-loss technique by the Institute of Polymer Materials (Friedrich-Alexander-University of Erlangen-Nürnberg). In the production process a curable liquid silicone rubber (LSR) mixture (Wacker Chemie AG, Munich, Germany) was spread evenly in a polytetrafluorethylene (PTFE) mould and salt particles of deliberately chosen grain size fractions were sprinkled onto the surface. Afterwards, the LSR was cured at 120 °C and the salt particles were rinsed off with water. This process generates similar surface textures as if the uncured silicone was pushed into a bed of granular salt, but allows an easier handling of the uncured LSR [26, 29].

The four textures included an untextured reference without pores (control), a fine texture (<63 µm pore size), a medium texture (>250 µm pore size) and a coarse texture (>500 µm pore size).

### Primary cell culture

Human primary fibroblasts (HFIB-D, cryo, provitro AG, Berlin, Germany) were cultivated in DMEM high glucose (Dulbecco’s Modified Eagle Medium, Sigma-Aldrich, Inc, St. Louis, MO, USA) and were supplemented with 10% FBS Superior (Foetal bovine serum superior, Biochrom/Merck, Berlin, Germany). Fibroblasts were then incubated at 37 °C under a humidified atmosphere of 95% air and 5% CO_2_. For all experiments, the cells were between passages 7 and 10. Medium was changed every second day.

### Seeding technique and study design

For each examination (d1 = day 1, d4 = day 4 and d7 = day 7) a separate 24-well plate was used. Triplicates were used for each of the four textures.

Round discs of 14 mm diameter were cut out of the silicone with a punch, steam-sterilized (via autoclave) and placed into a 24-well culture plate.

A steam-sterilized Teflon ring (625 mg) was placed on top of each disc to prevent floating and spinning and to enable the cells to adhere on the silicone discs. Subsequently, 500 µl of a cell suspension containing 8.0 × 10^4^ fibroblasts (in DMEM high glucose + 10% FBS superior) was seeded on top of the disc, inside the Teflon ring.

### WST-8 viability assay

To assess the cellular metabolic activity the Colorimetric Cell Viability Kit I (WST-8) was used according to the manufacturer’s instructions (PromoCell, Heidelberg, Germany). Experiments were performed after d1, d4 and d7. The medium was exchanged with 300 µl of DMEM high glucose and supplemented with 30 µl of the tetrazolium salt WST-8. Following 2 h of incubation, the absorption of 100 µl supernatant from each sample was measured at 450 nm with a reference wavelength of 600 nm (Multiskan GO; Thermo Fisher Scientific) [38].

### Staining

For DAPI staining, samples were transferred into a new 24-well culture plate after d1, d4 and d7, washed with phosphate-buffered saline (PBS) and fixed in 4% formaldehyde (Roti-Histofix 4%, Carl Roth GmbH Karlsruhe, Germany). Samples were washed with PBS and distilled H_2_O and stained with 1000 µl DAPI mixture (1 µg/ml) (Roche Molecular Systems, Pleasanton, CA, USA) per sample. After repeated washing with distilled H_2_O, they were stored with 200 µl PBS per sample at 4 °C.

### Microscopy

#### Inverted epifluorescence imaging

The images of the DAPI stained samples were taken with an inverted epifluorescence imaging system (Olympus IX83, CellSens software; Olympus, Tokyo, Japan). To track the adhesion and proliferation of the fibroblasts over time, overview pictures of the samples were taken after d1, d4 and d7.

#### Cell counting

A simple ImageJ algorithm was applied to estimate the overall cell count from DAPI epifluorescence overview images. After applying a 20 µm rolling ball filter to remove background artefacts, an intensity threshold is applied, using the Renyi Enthropy algorithm described by Kapur et al. [39]. A watershed filter is applied to the resulting binary image to separate overlapping nuclei and the *Analyze Particles* function of ImageJ is used to count particles within the expected size range of cellular nuclei from 50–100 µm^2^ [40].

#### Scanning electron microscopy (SEM)

Scanning electron micrographs (SEM, Zeiss Leica, Jena, Germany) of the silicone surfaces facilitated a detailed view of the distribution of pores and their variety in pore size and morphology. For SEM preparation, the samples were placed on metal stubs and sputtered with gold for one minute using a Turbo-Pumped Sputter Coater Q150T (Quorum Technologies, Laughton, United Kingdom). SEM analysis was then performed using a magnification of 500 fold.

#### Multi-photon microscopy

An upright version of the system described by Schneidereit et al. is used, applying the same mode-locked fs pulsed Ti:Sa laser (Chameleon Vision II, Coherent, Santa Clara, CA, USA) and Trimscope II system (TriMScope II, LaVision BioTec, Bielefeld, Germany) backbone but applying an upright microscopy stage with a CG FLuotar L25 × /0.95 W Visir objective (Leica, Germany) [41]. For excitation, a laser wavelength of 810 nm with an average power of 120 mW was applied while the label-free sample autofluorescence was acquired at 525 nm. 3D image stacks are recorded with typical voxel size of 0.4 × 0.4 × 3 µm and a field of view of 400 × 400 × 500 µm.

### Real-time quantitative polymerase chain reaction (qPCR)

Quantification of the mRNA expression of Collagen 1 (*COL1*), Collagen 3 (*COL3*), Transforming growth factor beta 1 (*TGFβ1*), Matrix metalloproteinase 2 (*MMP2*) and Tissue inhibitor of matrix metalloproteinase (*TIMP2*) was performed by qPCR. Total mRNA was isolated after d7 from all samples with a RNeasy micro kit (Qiagen GmbH, Hilden, Germany) according to the manufacturer’s protocol. To reverse transcribe the total mRNA into cDNA, a QuantiTect Reverse Transcription Kit was used (Qiagen GmbH, Venlo, Netherlands). Real-time quantitative PCR was performed using SsoAdvanced(R) Universal SYBR(R) Green Supermix (Bio-Rad Laboratories Inc., Hercules, CA, USA) and a Light Cycler (Bio-Rad CFX96 Touch™). Determined transcript levels were normalized to the housekeeping gene glyceraldehyde-3-phosphate dehydrogenase (GAPDH). Samples were tested as triplicates and evaluated by performing the 2^−^^ΔCt^ method. Used primer sequences are listed in Table 1.

**Table 1:** Primer sequences for qPCR

Gene	Forward primer (5’-3’)	Reverse Primer (3’-5’)
*GAPDH*	TCCACCCATGGCAAATTCCA	TTCCCGTTCTCAGCCTTGAC
*TGFβ1*	CATGGAGGACCTGGATGCC	TCCTGAAGACTCCCCAGACC
*COL1*	GCACCATCATTTCCACGAGC	AGTGGTTTGGATGGTGCCAA
*COL3 (A1)*	GGTGAAAGAGGATCTGAGGGC	AACACCACCACAGCAAGGA
*MMP2*	GCCGTGTTTGCCATCTGTTT	AGCAGACACCATCACCTGTG
*TIMP2*	TCTCGACATCGAGGACCCAT	TGGACCCAGTCGAAACCCTTG

### Statistics

For data comparison of the cell counting, a one-way ANOVA analysis (Sigma Plot, Systat Software) was applied and indicated with post hoc Tukey test. *p* < 0.05 was considered significant (*) and *p* < 0.01 was considered highly significant (**). Mean values of cell number were compared within same texture groups over the 3 examination time points and at the endpoint (d7) between texture groups.

The normal distribution for PCR and WST-8 was tested using the Shapiro-Wilk test. Hence, for data comparison of the PCR and WST-8, the non-parametric Kruskal-Wallis test (SPSS, IBM Deutschland GmbH, Germany) was applied on the four different textures. *p* < 0.05 was considered significant. In case of significant results, a Mann-Whitney-U test was performed.

## Results

### Viability of cells

Viability of cells was examined with the WST-8 assay. Cell viability increases over time on all three textures. Only the control (no texture) showed a consistent activity. Viability is highest on all examination time points for the medium texture followed by coarse and fine, but no significant difference could be found between the three examination days.

### DAPI overview images

**Fig. 1:**
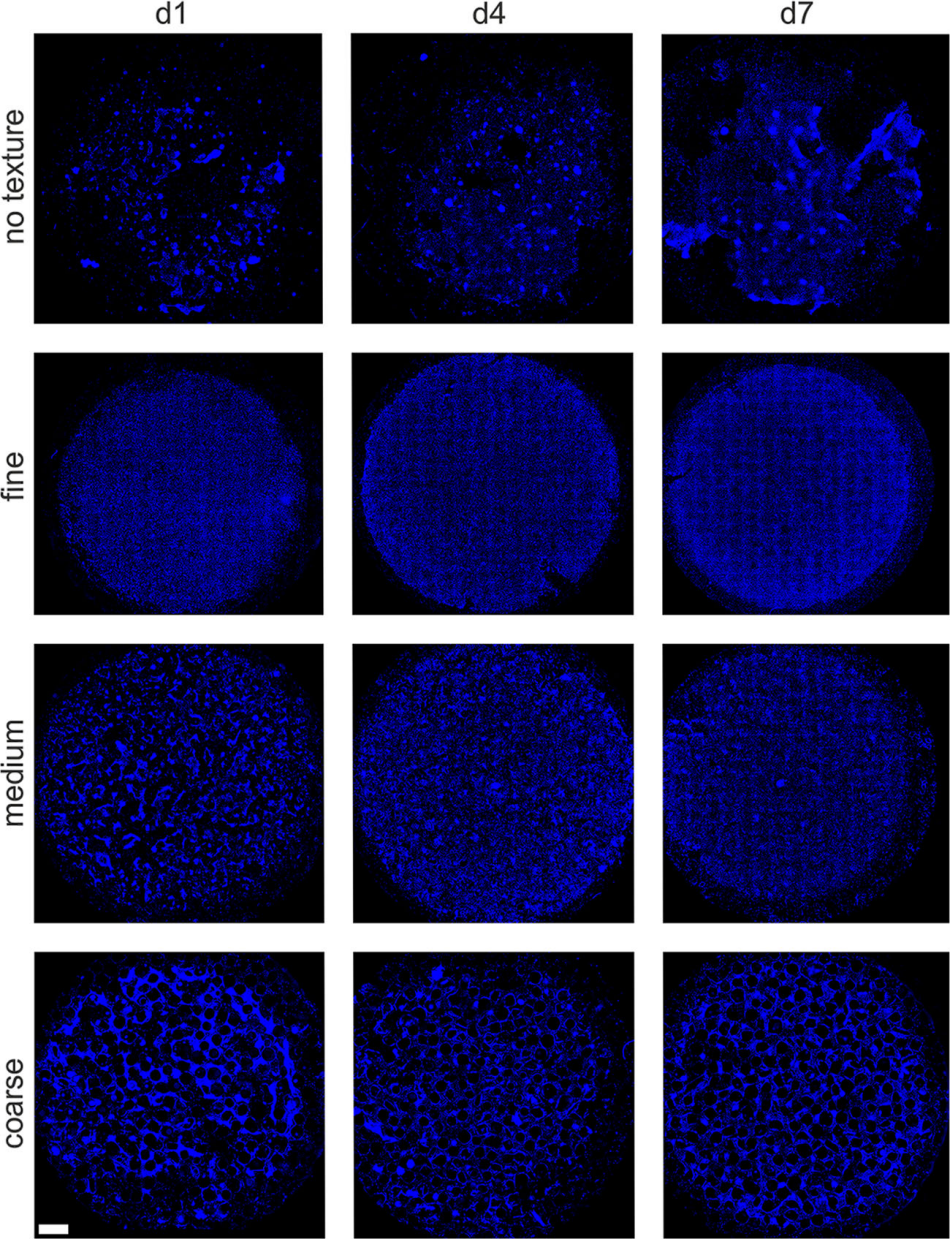
DAPI overview images of the different surface textures on d1, d4 and d7 (scale bar: 1 mm). Irregular cell distribution in the control (no texture). Dense cell growth in the fine and medium texture. Sponge-like cell growth in the coarse texture

The DAPI stained overview images show that the cells adhere, expand over time and spread on the silicone (Fig. 1). The control shows a noticeably more irregular, chaotic and less dense cell distribution. On the fine and medium texture, the cells grow densely on the surface and form a homogenous layer. On the coarse texture the ‘holes’ remain visible and the fibroblasts seem to grow entirely on the areas between the pores.

### Cell counting

A comparison on growth within same texture groups shows an increasing cell growth on all textures. Cell number of the control on d7 is significantly higher than on d1 and d4 (*p* < 0.01). The fine texture shows a decreasing numerical value on d7 with respect to d4. In the medium texture, cell number on d4 and d7 are significantly higher than on d1 (d4: *p* < 0.05, d7: *p* < 0.01). In the coarse texture d7 is significantly higher than d1 (*p* < 0.05).

A comparison of the growth endpoints (d7) between texture groups shows significantly higher cell numbers on fine and medium texture to coarse texture.

### SEM

**Fig. 2:**
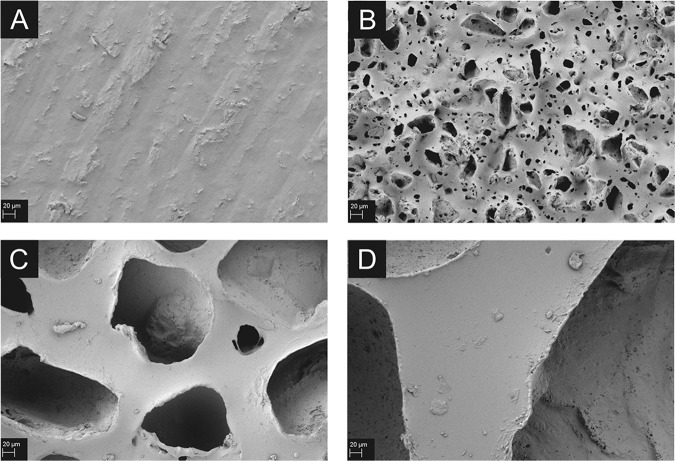
Representative scanning electron images of the 4 textures without fibroblasts, respectively, in 500-fold magnification (scale bar: 20 µm). Control (no texture) **a**, fine texture **b**, medium texture **c**, coarse texture **d**

To evaluate the pore structure and the surface generated by the salt-loss technique the samples were examined using SEM (Fig. 2). As described by Barr et al., the resulting implant surface remains “randomly-arranged, with cubical and sharp-edged cavities” [3]. Particularly, the fine texture shows a great variety in pore size. The SEM pictures revealed a marginal irregularity also for the non-textured control.

### Multi-photon microscopy

**Fig. 3:**
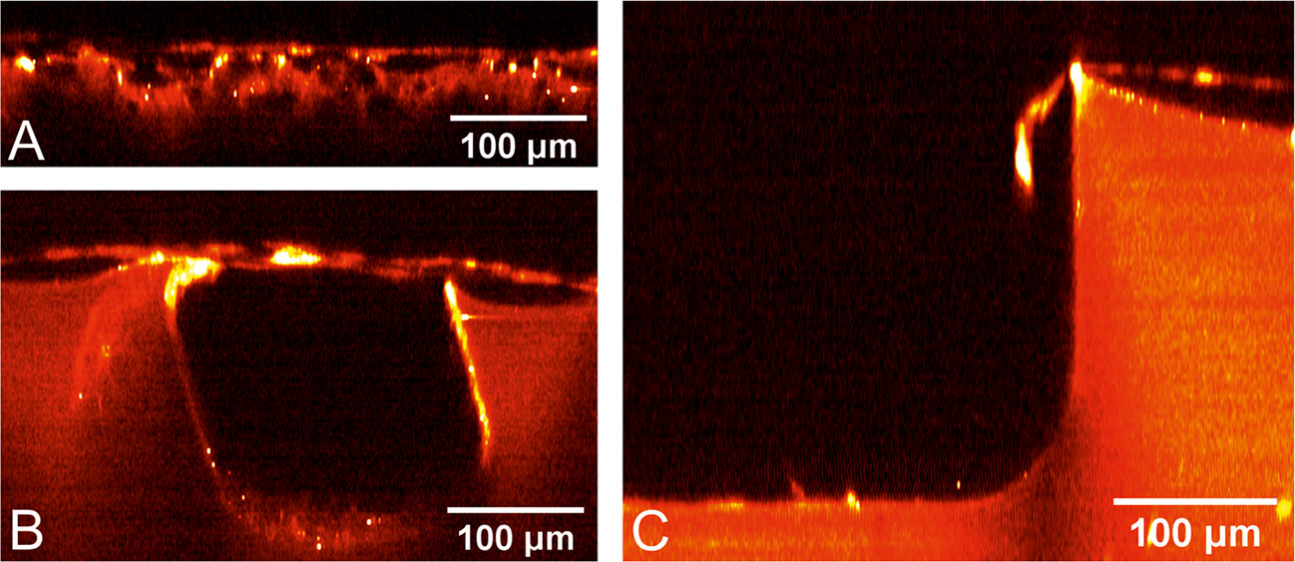
Representative multi-photon microscopy images of bridge-building fibroblasts on fine **a** and medium texture **b**; “Floating” fibroblasts on coarse texture **c**

The label-free visualisation of the cells using a multi-photon microscope scrutinized the morphology of the cells and their growth behaviour. Unexpectedly, instead of growing into the pores, the fibroblasts formed a bridge across them. The bridging depends on the pore size. In the fine and medium texture, the pores can be overgrown (Fig. 3a, b), though the distance in the coarse texture is too wide to be overcome by the cells. Figure 3c shows how the cells “fall” over the edge of the coarse pore and seem to float in the “air” when trying to grow over it.

### PCR

**Fig. 4:**
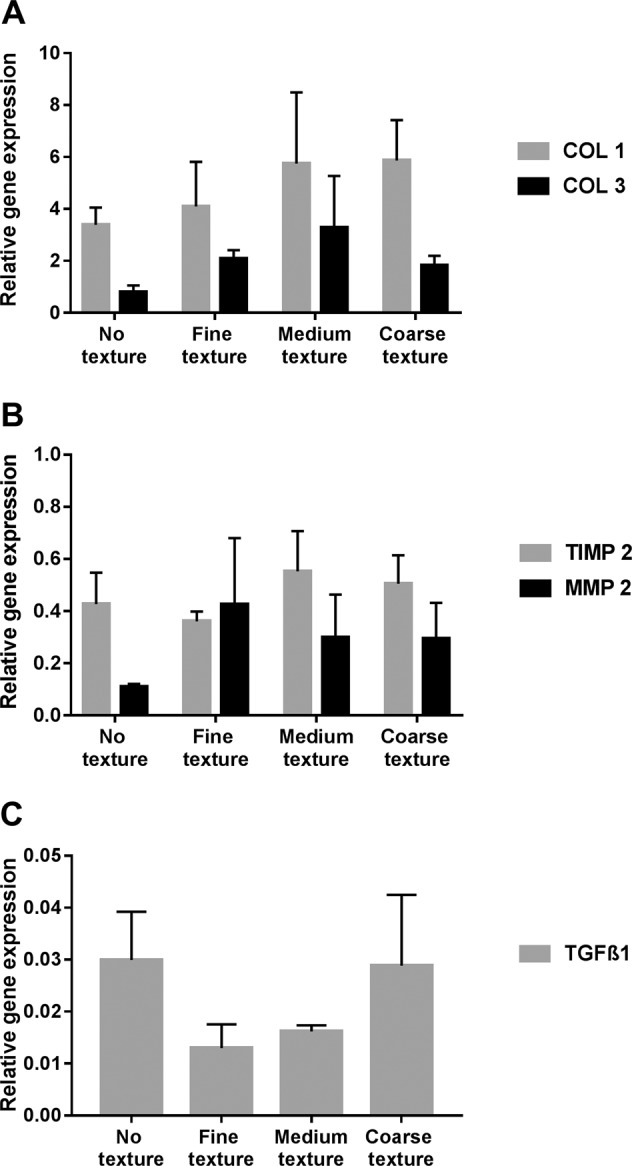
Gene expression 2^−ΔCt^ of *COL1*, *COL3* **a**, *TIMP2*, *MMP2* **b** and *TGFβ1* **c** in fibroblasts cultivated for seven days on different silicone textures. Highest values for *COL1* in medium and coarse, for *COL3* in the medium texture. Highest expression rate for *MMP2* in fine, *TIMP2* shows homogenous data. Highest values for *TGFβ1* in control and coarse texture

In the following, the PCR results of the 2^−ΔCt^ method are described. Figure 4 shows the gene expression for all aforementioned target genes. The analysis of the gene expression showed in all samples a very low mRNA expression for *TGFβ1*. No significance was found between the 4 groups.

The data of the gene expression analysis showed no statistically significant differences. The expression for *COL1* was highest in the medium and coarse texture. The highest expression rate for *COL3* was found in the medium texture, followed by the fine texture, coarse texture and control (no texture). For *MMP2*, the expression rate was higher in the fine texture than in any other texture, whereas medium and coarse textures were identical. *TIMP2* revealed in all textures homogeneous data.

## Discussion

Independent of the synthetic material or medical device, a foreign body reaction as a result of implantation has been well-known for decades. This reaction is ubiquitous and remains an unsolved problem until today, even though millions of different medical devices are being implanted in humans for various indications, and primarily without noteworthy serious side effects in most cases.

In the case of silicone breast implants, many approaches have been pursued to prevent the development of CC as a form of excessive foreign body reaction, in both clinical and experimental studies [42]. Besides possible preventive factors, like atraumatic tissue handling, aseptic conditions during the operation and placement of the implants either subpectorally or subglandularly, the surface of the implants and therefore, their interaction with human tissue after the implantation might play an important role. Consequently, implant surfaces have been modified using diverse techniques to create different kinds of textures containing different pore sizes and pore structures on a microscopical level. The salt-loss or sugar-loss technique as well as the method of imprinting using a negative contact imprint, e.g. polyurethane foam, are used among manufacturers today. All these techniques aim to improve the biocompatibility with a reduction of the foreign body reaction towards implanted materials. But the term biocompatibility is not clearly defined yet. Ratner promoted a more detailed look at this issue, as he differentiates between biocompatibility and biotolerability [43, 44]. Consequently, material that triggers normal tissue reaction regarding wound healing is defined as biocompatible, whereas biotolerable material rather leads to a low degree of inflammatory tissue response for long periods.

There is still insufficient knowledge on the impact of the surface structure on CC at the cellular level [45]. It remains unclear how different surfaces, with irregular pore distribution, pore depth and different contact angles, influence the surrounding cells, even if studies exist that showed little fibrosis in materials with smaller sized pores [29, 31, 37, 46–50]. It is already known that cells are guided by their surrounding topography, and it is suggested that they are “spatially aware”. Cells might therefore probe their adjacent surroundings leading to an interaction which could down-stream cell reactions to biomaterials or the extracellular matrix (ECM) [51]. Regular surface characteristics of textured surfaces might promote the growth of fibroblasts on the surface resulting in a decrease of contractile forces. This contact modification could result in a reduced fibrotic activity around implants [37, 52].

Because this topic has been of high relevance for years, and a possible association between the implant surface structure and BIA-ALCL represents a cutting-edge research topic, we performed this study. We directly compared, to the best of our knowledge, for the first time, four deliberately modified different surface textures of rubber silicone material with their respective different pore sizes on a cellular level. Therefore we focused on the salt-loss technique and different intervals of pore sizes.

Since fibroblasts play a major role in fibrosis development, we focused on the interaction between this type of cells and the silicone material [45, 53–58]. The most important markers related to CC known today were assessed [59]. The gene expression rates for *TGFβ1* as a leading marker for fibrosis and *COL1* and *COL3* were analysed. *MMP2* and *TIMP2*, which play an important role in the remodelling process of the ECM and therefore in the regulation process of wound healing and foreign body reaction, were used [60–64].

Furthermore, we assessed the cell distribution pattern and cell adhesion as well as the cell growth in relation to the pore size.

DAPI staining was used for analysis of distribution pattern of the fibroblasts. A homogenous and dense cell growth could be detected on fine and medium textures, whereas the control (no texture) showed irregular cell growth and multiple spots without cells. This finding is similar compared with the coarse texture, but is due to the large pore size in the latter one, in particular corroborated by the multiphoton images revealing a failure of fibroblasts to ‘bridge’ the larger gaps in the coarse material with larger pore sizes. The WST-8 cell viability assay after 7 days is in accordance with the results of the DAPI overview staining by showing the highest cell viability for the medium texture. The cell counting revealed highest cell number in the fine and medium texture, coming along with the aforementioned results. But as a limiting finding the development of the absolute cell count within one experimental group and during the experimental course represented divergent from the WST-8 assay. This might be due to a methodical difference between the WST-8 assay and the algorithm used for absolute cell counting. Whereas the WST-8 assay reliably detects vital cells, the absolute cell counting was performed on the basis of the DAPI staining with non-vital cells. The multi‐photon laser scanning microscope revealed cell growth in the fine and medium textures as an almost closed layer over the silicone pores building cell bridges. No single cells could be detected in the pores using them for ideal cell-size dependent ingrowth, as one could hypothesis an ideal pore-size/cell-size relation to be a preventive factor for exceeding foreign body reaction. In the coarse texture samples cells dropped into the pores or showed an inhomogeneous growth pattern along the pores with finger-shaped cell extensions and missing intercellular contact. Therefore, fine-pored textures seem to enable homogenous cell growth, even if the cells do not ideally fill the pores by their cell body. Hence the induction of a best possible physiological cell response might positively influence and therefore reduce the foreign body reaction. Furthermore, the hypothesis is underlined that fibroblasts need a sort of irregular surface of synthetic or artificial materials for an uncomplicated integration process. On the other hand, large-pored surfaces might lead to a more inhomogeneous and uncontrolled cell growth and thus, to an increased fibrotic activity.

During the physiological wound healing process, as well as in the course of breast capsule formation, the differentiation of fibroblasts to myofibroblasts plays an important role. This process is initialized by the *TGFβ1* signal pathway. *TGFβ1* is well described as main molecule in the formation of CC [65, 66]. As a cytokine that influences cell proliferation and differentiation, it as well represents a potent fibrotic, angiogenetic and inflammatory mediator and therefore, plays a major role in fibrotic diseases. A low or decreased expression of *TGFβ* is associated with a reduced fibrotic tendency. Former studies tried to inhibit the *TGFβ1* pathway in order to reduce the development of myofibroblasts and with it the formation of CC [67, 68].

In our preliminary study, gene expression analysis revealed a trend for decreased fibrotic activity for the fine and medium texture. *TGFß1* showed the lowest expression for those groups. Additionally *COL3* and the relation of *COL1* to *COL3* affirmed this observation, as these results are known for reduced fibrotic response [69].

The role of *MMPs* and *TIMPs* in the context of activation or inhibition in the development of CC is not thoroughly understood. The degradation and collagenous remodelling could be a key process in fibrosis. *TIMPs* are endogenous inhibitors of *MMPs* and four homologous subtypes are known (*TIMP-1*, *2*, *3* and *4*). In general, every *TIMP* is capable of inhibiting all *MMPs*. Only the efficacy of *MMPs* inhibition varies for each *TIMP*. Related to our study, *TIMP2* inhibits metalloproteinases, including *MMP2*, but is also required for *MMP2* activation [70]. Moreover, *MMP2* is said to have an antifibrotic effect in ECM remodelling and an inhibitory activity against *COL1* [71]. As seen for liver fibrosis, *TIMPs* seem to have divergent roles and do not appear to function strictly by blocking the matrix-degradation or the collagenolytic activity of metalloproteinases [71].

In this study, *MMP2* was found to be expressed highest in the fine and medium texture. Together with the results of *TGFß1*, *COL1* and *COL3* this could be interpreted as an active remodelling process towards a more controlled cell reaction against the silicone surfaces and confirm the antifibrotic effect of *MMP2*. *TIMP2* showed no remarkable differences between the single surfaces. Ulrich et al. assessed tissue from women with CC of smooth and textured implants (negative-contact polyurethane foam imprinting). They found an upregulation of *TIMP1* and *TIMP2* in relation to the graded severity of the CC. Furthermore, they observed a significantly higher expression of both genes in smooth implants [64]. Kyle et al. found an upregulation of *TIMP4* in contracted breast capsule explants in smooth and textured implants [72]. Therefore, the interpretation of our results in this context seems difficult due to the different roles of *TIMP*.

In general, interactions of cultured cells with biomaterials can vary depending on multiple conditions concerning the material characteristics, cell type, cell age, culture conditions etc.—as is known from similar experience from tissue engineering and regenerative medicine [73–76]. Furthermore a limitation is that cell culture experiments might exceed 7 days. But crucial changes and cell reactions related to the assessed cells take place in an early stage. Moreover further studies could assess different pore sizes of alternative silicone surfaces.

Nevertheless, our findings are suitable to shed more light on the behaviour of biocompatible silicone materials and the interaction of cells with various implant surfaces. To further clarify the various cell-cell interactions, future studies shall take into consideration that different cell types may interact differently with silicone breast implants, and thus could be differently involved in the foreign body reaction. Consequently, further experiments in this context could focus on co-culture settings using connective tissue and breast tissue for the best possible imitation of an in vivo setting (and directly analyze ECM formation and activation of pathways on the protein level). Moreover, modifications of other surface textures should be assessed to gain more insights into the influence of distinct texture pore sizes in relation to the different existing texturing techniques.

## Conclusion

Besides multiple possible factors, the surface texture of silicone breast implants seems to have a major impact on the development of capsular contracture. Different existing implant texturing techniques with different pore sizes may exert a distinct influence on the foreign body reaction and cell adherence. For surfaces produced with the salt-loss technique we were able to show an antifibrotic effect of smaller-sized pores in an in vitro model using human fibroblasts in this study. These findings underline the hypothesis of a key role of the implant surface and the pore size and pore structure in preventing capsular contracture.
